# Naturally Derived SENP1 Inhibitors with Anticancer Activity

**DOI:** 10.3390/ijms262211210

**Published:** 2025-11-20

**Authors:** Renata Krupa, Katarzyna Woźniak

**Affiliations:** Department of Molecular Genetics, Faculty of Biology and Environmental Protection, University of Lodz, Pomorska 141/143, 90-236 Lodz, Poland; renata.krupa@biol.uni.lodz.pl

**Keywords:** SENP1, sumoylation, momordin Ic, hinokiflavone, triptolide, ursolic acid, streptonigrin, vialinin A, thelephantin G

## Abstract

SENP1 (sentrin-specific protease 1) mediates sumoylation, a reversible post-translational modification that attaches the SUMO (small ubiquitin-like modifier) protein to target proteins. These modified proteins are essential in many key cellular processes, including cell cycle regulation, DNA repair, and apoptosis. Disruptions in the balance between sumoylated and desumoylated proteins can lead to various pathological conditions, such as cancer. Experimental data suggest that certain natural compounds, including momordin Ic (Mc), hinokiflavone (HNK), triptolide (TPL), ursolic acid (UA), streptonigrin (SN), vialinin A (VA), thelephantin G (TG), and others, effectively inhibit SENP1 activity, thereby influencing the levels of sumoylated proteins and cellular processes. This article reviews existing knowledge on the structure and function of natural SENP1 inhibitors, particularly their potential application in cancer therapy, including their capacity to overcome resistance to conventional chemotherapies. Some of the natural SENP1 inhibitors tested so far interact directly with the enzyme’s active site. The current understanding of how this interaction occurs is also discussed.

## 1. Introduction

Sumoylation, one of the post-translational modifications of proteins, is a reversible process of attaching SUMO (small ubiquitin-like modifier) proteins, approximately 11 kDa in size, to target proteins. It affects, among other things, the regulation of DNA repair, immune response, the progression of the cell cycle, and apoptosis. Post-translational modifications of proteins can alter their conformation, hydrophobicity, charge, or stability, thereby affecting the functions they perform [1,2]. Four isoforms of the SUMO protein (SUMO-1–4) are present in mammals, and all show a high degree of similarity. Due to the approximately 97% homology between the SUMO-2 and SUMO-3 proteins, they are often referred to as the SUMO-2/3 protein. The SUMO-4 protein has another amino acid, proline (Pro), in the place where Gln90 is present in other SUMO proteins. This change in the structure of SUMO-4 prevents the SENP from accessing the appropriate areas of the protein precursor, and consequently, it is unable to produce its mature form [3]. Additionally, SUMO-1–3 proteins are found in all human tissues, whereas SUMO-4 mRNA has been localized to the kidney, lymph nodes, and spleen [1].

**Figure 1:**
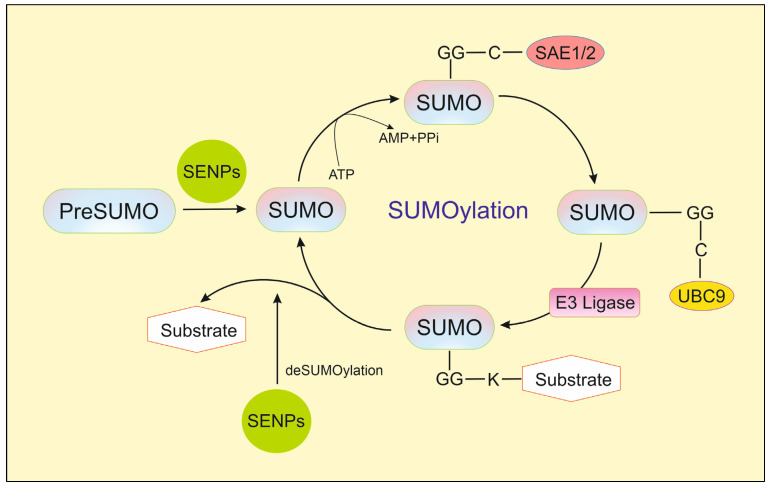
The sumoylation cycle [5].

SUMO proteins are synthesized as precursors. An important site in SUMO proteins is the C-terminus, from which four amino acids are cleaved by sentrin-specific proteases (SENPs) to expose two glycine residues. They will covalently bind to the target protein at the lysine site. SENPs are not only involved in the maturation of precursors but also play a crucial role in desumoylation, i.e., reversing the modification of the target protein. Another group of proteins involved in sumoylation are enzymes: activating E1, conjugating E2, and E3 ligases [4]. These enzymes are responsible for carrying out the individual stages of the sumoylation cycle. The first stage of sumoylation involves the maturation of the SUMO protein, which is facilitated by the SENP (Figure 1). Then, the SUMO protein binds to the E1 enzyme. This enzyme consists of two subunits: SAE1 and SAE2 (SUMO-activating enzymes). Using energy from ATP, a thioester bond is formed between cysteine (Cys173) in SAE2 and glycine (Gly97) in the C-terminus of the SUMO protein. The SUMO protein is transferred from E1 to the E2 enzyme, also called UBC9. A thioester bond is formed between Gly97 of SUMO and Cys93 of the UBC9 protein. UBC9 protein exists in only one form and interacts equally well with SUMO-1 and SUMO-2/3. In vitro, the sumoylation process may terminate with the action of UBC9, where the C-terminus of SUMO is connected to the target protein via an isopeptide bond in place of a lysine. However, in vivo, the action of an E3 ligase is still required to strengthen the connection in the modified protein. The largest group of SUMO E3 proteins consists of PIAS proteins (protein inhibitors of activated signal transducer and activator of transcription), (PIAS 1-4) [4]. SENP proteins are required for desumoylation. To date, six SENPs (1, 2, 3, 5, 6, and 7) have been identified and classified into subfamilies based on their interactions with specific SUMO proteins [1]. The first are SENP1 and SENP2, which act on SUMO-1 and SUMO-2/3 proteins. Additionally, SENP1 is involved in the maturation of all SUMO precursors through its hydrolase activity [4]. The second subfamily comprises SENP3 and SENP5 proteases, which separate only SUMO-2/3. The third subfamily, which includes SENP6 and SENP7, also affects only SUMO-2/3, as only they form polymers, and it is mainly these polymers that are detached by these proteases. The key element of the structure of SENPs is the catalytic domain composed of cysteine, histidine, and asparagine, which is present at the C-terminus, where the presence of about 250 amino acid residues determines the possibility of deconjugation of specific SUMO proteins. Since sumoylation and desumoylation play crucial roles in cellular processes such as cell proliferation, DNA repair, apoptosis, and gene expression regulation, an imbalance between them can lead to numerous errors in cellular function and, consequently, result in various diseases. Too high or too low activity of individual enzymes of the sumoylation cycle can contribute to the formation of many types of cancer, diabetes, or heart disorders [4].

## 2. SENP1

SENP1 is one of the most essential SENPs, with a well-established role in carcinogenesis [5,6,7]. It is a cysteine protease composed of 643 amino acids with a molecular weight of 73 kDa, and its gene is located on chromosome 12q13.1. SENP1 includes a characteristic catalytic triad His-Asp-Cys at the C-terminal. *SENP1* is overexpressed in lung cancer [8], glioma [9], liver cancer [10], and other cancers [7]. The high expression of *SENP1* is significantly correlated with an adverse prognosis in various cancers, including ovarian cancer [11], osteosarcoma [12,13], and colorectal cancer [14]. Moreover, it affects the sensitivity of cancer cells to chemo- and radiotherapy [15,16]. It has been demonstrated that knocking out the *SENP1* gene or inhibiting SENP1 activity prevents the proliferation, cell cycle progression, invasion, and migration of cancer cells [7]. Therefore, inhibiting SENP1 activity could be a new strategy for defeating cancer cells, including those resistant to standard treatments [17,18]. In recent years, many molecules with SENP1 inhibitor activity have been developed and tested [19,20]. In addition to synthetic SENP1 inhibitors, natural inhibitors of this enzyme are sought [7,21].

## 3. Natural Inhibitors of SENP1

### 3.1. Momordin Ic

**Figure 2:**
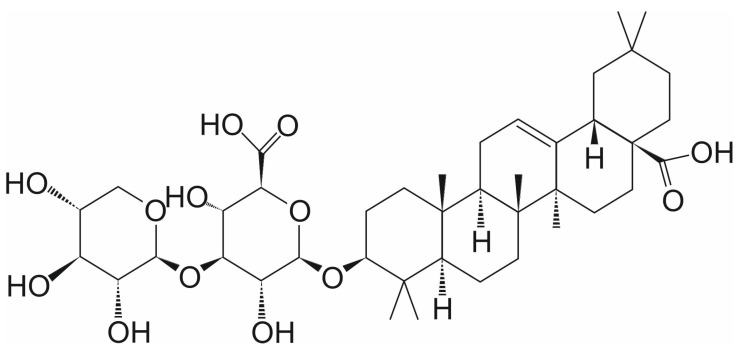
The chemical structure of momordin Ic (Mc).

Momordin Ic (Mc) (3-O-(β-D-Xylopyranosyl(1→3)-β-D-Glucopyranosiduronic Acid) Oleanolic Acid) is a pentacyclic triterpenoid from the group of saponins, which is found in many plants used in natural medicine, e.g., *Kochia scoparia* (L.) [22] (Figure 2). Mc is an effective agent against many types of cancer, including prostate [23], liver [24,25,26,27], and colorectal cancer [28]. It effectively inhibits SENP1 activity with an IC_50_ of 15.37 µM in prostate cancer cells [23]. The cellular thermal shift assay (CETSA) was used to investigate whether Mc directly binds to SENP1. CETSA is based on a biophysical principle that is ligand-induced thermal stabilization of target proteins. As shown in the first thermal shift test with purified SENP1, Mc contributed to a significant reduction in SENP1’s thermal stability. Additionally, it reduced protease accumulation in a dose-dependent manner. These results showed a change in the thermal stability of SENP1. The second drug affinity responsive target stability (DARTS) assay confirmed that Mc protects the SENP1 from pronase-induced proteolysis. This indicated a direct interaction between SENP1 and Mc [23].

In prostate cancer PC-3 cells, Mc contributed to an increase in SENP1 accumulation proportionally to the Mc concentration. These results conclude that Mc directly interacts with SENP1. By inhibiting SENP1 activity, Mc contributes to the reduction in prostate cancer cell proliferation. The overexpression of *SENP1*, which is common in cancer cells, results in a decrease in the amount of sumoylated proteins, but this effect is partially reversed by the administration of Mc. Mc contributes to an increase in the number of cells in the G1 and G2/M phases. The PE-Annexin-V/7AAD staining assay showed an increased number of apoptotic cells. However, *SENP1* overexpression negates the effects of proliferation inhibition, apoptosis induction, and cell population growth in the G1 phase [23]. In vivo studies have also shown the high effectiveness of Mc in inhibiting prostate cancer. These studies were conducted by subcutaneously implanting PC-3 cells into BALB/c mice. When the tumors were palpable, daily intraperitoneal Mc was started at 10 mg/kg. After 20 days, the tumors in mice given Mc were smaller than in the control group, indicating the anticancer effect of this compound [23].

In studies of Mc effect on colon cancer, interesting results were obtained on the HCT-116 and HCT-8 cell lines [28]. Mc reduced the ability to form colonies and decreased the viability of these cells. Moreover, it was demonstrated that Mc induced cell cycle arrest and apoptosis, thereby inhibiting the growth of colon cancer cells. Molecular evidence demonstrated that Mc exerted anti-growth activity in colon cancer cells by inhibiting the SENP1/c-Myc signaling pathway. SENP1 is essential for desumoylation of c-Myc, which increases its stability and activity [29].

Wang et al. reported that Mc induces apoptosis in HepG2 liver cancer cells through oxidative stress-regulated mitochondrial dysfunction involving MAPK and PI3K-mediated iNOS and HO-1 pathways [24]. Next, Wang et al. demonstrated that PI3K and MAPK-dependent PPARγ activation are involved in Mc-induced apoptosis in HepG2 cells [25]. Moreover, Wang et al. demonstrated that Mc effectively prevented the attachment, migration, and invasion of HepG2 cells by activating PPARγ and inhibiting COX-2 [26,27]. Additionally, it was shown that Mc simultaneously induced apoptosis and autophagy by activating reactive oxygen species (ROS)-related PI3K/Akt, MAPK, and NF-κB signaling pathways in HepG2 hepatoblastoma cancer cells [30]. In another cell type, specifically HaCaT cells (a human immortalized epidermal cell line), Mc inhibited proliferation via the Wnt/β-Catenin pathway [31]. Recently, it was demonstrated that the combination of Mc with gallic acid, both enriched in the extract of *Momordica charantia*, significantly eliminates both normoxic active and hypoxic dormant tumor cells, representing a promising lead in anticancer drug development [32].

Research suggests that Mc could also help overcome cancer resistance. It was found that SENP1 directly controls JAK2’s localization through desumoylation. The SENP1/JAK2 pathway is active in cisplatin-resistant ovarian cancer and is dependent on the transcription factor RUNX2. When RUNX2, SENP1, and JAK2 are active, they play a key role in platinum resistance. Using Mc or SENP1 siRNA to inhibit SENP1 shows a strong synergistic effect with cisplatin in treating platinum-resistant ovarian cancer [33].

Sirtuin 3 (SIRT3) deacetylase is a key regulator of chemoresistance in acute myeloid leukemia (AML) cells. It can modulate mitochondrial metabolism and ROS levels. SENP1 desumolates SIRT3, which enhances its deacetylase activity. Thus, deregulation of SIRT3 sumoylation may increase chemoresistance in AML. On the other hand, inhibition of SENP1 activity may inactivate SIRT3 and sensitize AML cells. Therefore, Mc may have therapeutic potential for overcoming resistance in AML resulting from SIRT3 activity regulated by sumoylation [34].

### 3.2. Hinokiflavone

**Figure 3:**
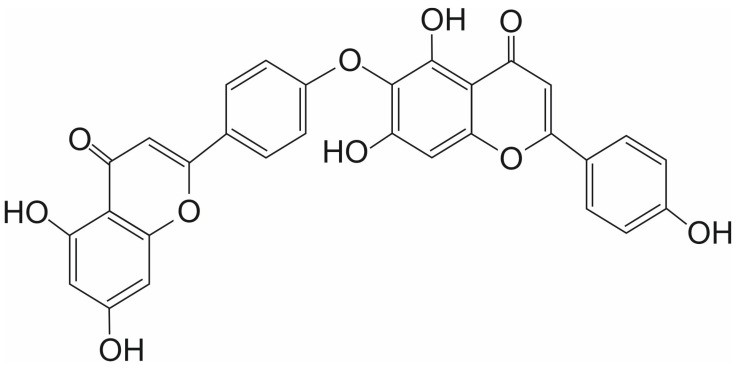
The chemical structure of hinokiflavone (HNK).

Hinokiflavone (HNK) is a biflavonoid discovered in 1958 in Japan. It was first isolated from the dried leaves of the plant *Chamaecyparis obtusa* Endlicher (also known as Hinoki cypress, Japanese false cypress) [35] (Figure 3). HNK, belonging to the biflavonoids of the C-O-C type, has anticancer activity. It is active against various cancer cell types, including colon, hepatocellular, breast, and nasopharyngeal cancers [35]. HNK and other bioflavonoids can inhibit the activity of MMPs (matrix metalloproteinases) a class of zinc-dependent peptidases that remodel the extracellular matrix, thereby favoring tumor invasive processes. Inhibition of MMPs, mainly MMP-2 and MMP-9, is a key element of the mechanism of anticancer activity of HNK. HNK can form stable complexes with MMP-9 via interactions with its catalytic active site. Molecular modeling predicted that HNK can bind deeply into the active site cavity of MMP-9, primarily through three hydrogen bonds with the NH groups of Gly-215, Tyr-423, and the C=O group of Glu-402, and hydrophobic contacts with several amino acids [36].

HNK suppressed colorectal tumor cell proliferation, induced apoptosis via ROS-mitochondria-mediated apoptotic pathway, and inhibited tumor cell migration and invasion. The antitumor activity of HNK was also validated in a mouse model, with no observed toxicity [37]. It also induces apoptosis in hepatocellular carcinoma (HCC) and breast cancer cells (MDA-MB-231) [35]. Recently, HNK was identified through virtual screening as a ligand that binds the MDM2-MDMX RING domain and inhibits MDM2 E3 ligase activity [38]. MDM2 and its homolog MDMX are the key negative regulators of p53. MDM2 acts as the E2 ubiquitin ligase targeting p53 for ubiquitination-mediated proteasomal degradation. This protein is frequently overexpressed in human cancers. HNK acting as a down-regulator of MDM2 and MDMX could be a potential anticancer agent in cancers with *MDM2* overexpression [38].

Interesting results on the anticancer potential of HNK were also provided by studies of Pawellek et al. [39]. These studies, as shown by the DARTS and CESTA assays, demonstrated that HNK can interact directly with SENP1. In cells incubated with HNK, an increase in polysumolated proteins was observed. Interestingly, it was shown that HNK can alter the alternative splicing of the MCL1 (Myeloid Cell Leukemia 1) pre-mRNA, leading to the synthesis of the short pro-apoptotic isoform MCL1-S rather than the long anti-apoptotic isoform MCL1-L. The longest form of MCL1, MCL1-L, prevents apoptosis by inhibiting the action of proteins that activate the mitochondrial apoptosis pathway. In contrast, the shorter forms of this transcript, MCL1-S, lack a hydrophobic groove. This groove typically interacts with the BH3 domain of pro-apoptotic proteins. Without it, the shorter forms do not inhibit apoptosis [40].

Recently, it was observed that HNK had antiproliferative effects and promoted the apoptosis of cisplatin-resistant bladder cancer cells [41]. Mechanistically, HNK inhibited CK2α activity, resulting in reduced phosphorylation of key proteins involved in survival signaling (Akt, Stat3) and DNA repair (XRCC1). HNK also downregulated the expression of base excision repair genes, including *MUTYH*, *OGG1*, and *XRCC1*. Additionally, HNK enhanced the cytotoxic effects of chemotherapeutic agents such as doxorubicin and mitomycin C. Collectively, HNK targets both survival and DNA repair pathways, supporting its potential as a therapeutic and chemosensitizing agent in cisplatin-resistant bladder cancer [41]. Other studies also suggest that the deregulation of sumoylation in DNA repair proteins, including the inhibition of SENP1 activity, may be a promising new strategy for sensitizing cancer cells to chemotherapy [42,43].

### 3.3. Triptolide

**Figure 4:**
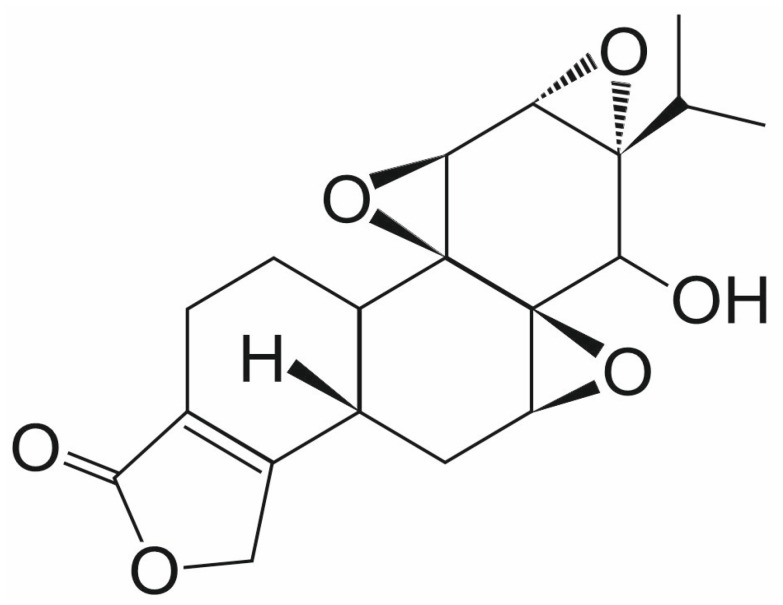
The chemical structure of triptolide (TPL).

Triptolide (TPL) is a natural diterpenoid epoxide that is extracted from the Chinese herb *Tripterygium wilfordii* (Figure 4). TPL exhibits anticancer activity against many human cancers, including prostate cancer, leukemia, and glioma [44,45,46]. In the NCI-60 screen, TPL demonstrated activity against all lines, with IC_50_ values ranging from 2.6 to 103 nM [47]. Based on the recent preclinical investigations, TPL is linked to the induction of death of cancer cells by triggering cellular apoptosis via inhibiting heat shock protein expression (HSP70) and cyclin-dependent kinase (CDK) by upregulating the expression of P21. MKP1, histone methyltransferases, and RNA polymerases have all recently been identified as potential targets of TPL in cells. Autophagy, AKT signaling pathway, and various pathways involving targeted proteins such as A-disintegrin and metalloprotease-10 (ADAM10), Polycystin-2 (PC-2), dCTP pyro-phosphatase 1 (DCTP1), peroxiredoxin-I (Prx-I), TAK1 binding protein (TAB1), kinase subunit (DNA-PKcs), and the xeroderma-pigmentosum B (XPB or ERCC3) have been exploited [48]. Moreover, TPL is responsible for enhancing the effectiveness of various chemotherapeutics, including cisplatin, carboplatin, PI3K inhibitors, and anthracyclines [49,50,51].

Huang et al. demonstrated that TPL inhibited cell growth and induced cell death in prostate cancer cell lines, including LNCaP and PC-3 [44]. The cell viability assay revealed significant inhibition of cell proliferation, even at very low concentrations of TPL, specifically 5 nM for LNCaP cells and 10 nM for PC-3 cells. TPL also significantly inhibited the growth of xenografted PC-3 tumors in nude mice. TPL down-regulated *SENP1* expression on both mRNA and protein levels in dose-dependent and time-dependent manners, resulting in an enhanced cellular sumoylation in prostate cancer cells [44].

No experimental data confirm the direct interaction of TPL with SENP1; therefore, the reduction in *SENP1* expression may result from the inhibition of other genes, such as *AR* (androgen receptor). AR is sumoylated at lysine residues 386 and 520. Mutation of these residues enhances the transactivation ability of AR, suggesting that sumoylation plays a role in regulating AR activity. Moreover, four AR coregulators, SRC-1, SRC-2/GRIP1, p300, and HDAC1, have also been found to be sumoylated. Mutating two sumoylation sites in *HDAC1* significantly reduced HDAC1-mediated transcriptional repression. Both AR and HDAC1 were targets of SENP1; however, the effect on AR-dependent transcription was primarily mediated by HDAC1 desumoylation. SENP1 could overcome the HDAC1 repressive function and reduce HDAC1 deacetylase activity. Thus, the data strongly support the role of SENP1 as a novel activator of AR-dependent transcription through desumoylation of HDAC1 [44,52]. Suppression of *SENP1* expression and decreased activity of desumoylation on AR and histone deacetylase 1 (HDAC1) in PCa enhances AR sumoylation and deacetylation by HDAC1, leading to inhibition of AR-mediated transcription. TPL could inhibit *AR* expression directly, inducing suppression of AR-mediated transcription. *AR* down-regulation also inhibits *SENP1* expression, enhancing the cellular sumoylation activities. TPL could directly inhibit *c-Jun* expression, thereby reducing AP-1 and c-Jun-mediated transcription. The non-transcriptional function of c-Jun as a co-activator was also suppressed. Furthermore, TPL could influence the expression of other genes or target other proteins to disrupt the abnormal functions of these genes or proteins in PCa. As a result, inhibition of AR and c-Jun-mediated transcription, suppression of other target functions by SENP1 desumoylation, and interruption of other important molecular functions contribute to inhibiting PCa proliferation and progression, facilitating apoptosis [44].

The clinical applications of TPL are limited due to its poor water solubility and severe cytotoxicity. To overcome these limitations, various TPL derivatives and drug delivery systems, particularly nanocarriers, have been developed. Furthermore, various preclinical and clinical studies have demonstrated that TPL and its derivatives exhibit excellent antitumor effects by targeting proteins involved in multiple signaling pathways [51]. For example, Minnelide is a more water-soluble synthetic prodrug of TPL, which is converted to TPL in vivo [53]. Following promising activity observed in a Phase I trial, a Phase II International open-label clinical trial of Minnelide in patients with chemotherapy-refractory metastatic pancreatic cancer (MinPAC) (NCT03117920) is currently in progress [54]. Glutriptolide, a glucose conjugate of TPL with improved solubility and reduced toxicity, demonstrated tumor control in vivo, likely due to the sustained, stepwise release of active TPL within cancer cells [55]. A second-generation glutriptolide has been recently reported for targeting hypoxic cancer cells with increased glucose transporter expression [56].

### 3.4. Ursolic Acid and Its Analogs

**Figure 5:**
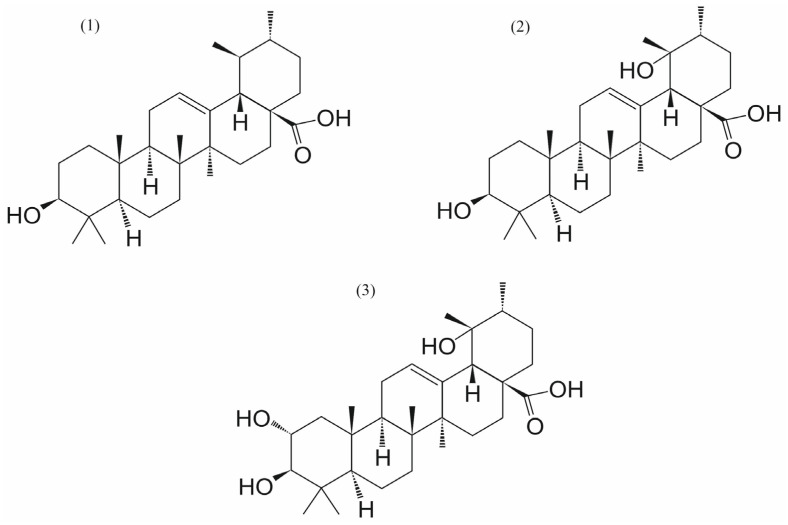
The chemical structures of ursolic acid (UA) (**1**) and its analogs: pomolic acid (**2**) and tormentic acid (**3**).

Ursolic acid (UA) is a pentacyclic triterpenoid that has numerous biological properties, such as anticancer, antidiabetic, antiarrhythmic, antihyperlipidemic, antimicrobial, antihypercholesterolemic, and anticardiovascular [57] (Figure 5). It is found in plants such as rosemary, marjoram, and oregano, as well as in apple peel. Various UA analogs were synthesized through modification at positions C2-OH, C3-OH, and C17-COOH [58]. The C-17 amide and amino analogs of UA showed greater anticancer activity than the parent compound [58].

**Figure 6:**
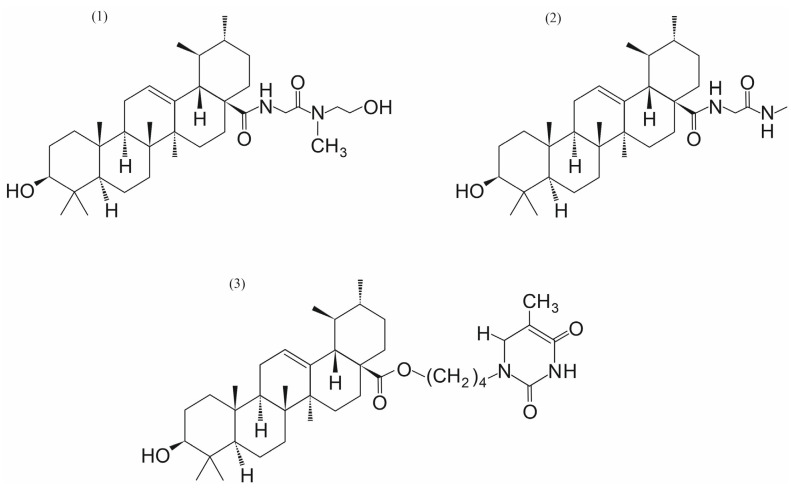
UA derivatives with anticancer activity. UAMMC9 (**1**) [16], compound (**2**) [18], and the hybrid of UA with uracil derivative (**3**) [60].

IC_50_ of cytotoxicity for UA for cancer cell lines varies between the concentrations of 20.6 µM and 65.0 µM [59]. The screening identified UA that re-sensitized resistant cells to cisplatin with a lower IC_50_ than TPL (UA = 0.86 μM; TPL = 1.37 μM), suggesting its good synergy with cisplatin. The IC_50_ of UA (0.24 μM) is much lower than Mc (31.76 μM) in IGROV1 CR cells (ovarian cancer cells resistant to cisplatin) [16]. An in vitro desumoylation assay confirmed the inhibition of SENP1 by UA, with an IC_50_ of 0.0064 μM, compared to 19.91 μM by Mc. CETSA revealed that at higher temperatures, the SENP1 protein was stabilized in cells treated with UA, showing a direct interaction between SENP1 and UA. Molecular docking showed that UA entered the hydrophobic catalytic cleft of SENP1. This analysis demonstrated that the 3-hydroxyl group of UA forms two hydrogen bonds with key catalytic residues Cys603 and Ser601. The 28-carboxyl group was located at the opening of SENP1 and formed an extra hydrogen bond with His529. Hydrophobic residues Trp465, Leu466, Ile471, Val532, and Trp534 created a hydrophobic cleft, which can interact with the pentacyclic triterpenoid scaffold. The potential medical use of UA is limited by its poor aqueous solubility, rapid metabolism, and low bioavailability. Zhang et al. synthesized eight derivatives (UAMMC1-7 & 9) of UA by introducing various hydrophilic groups to a 28-carboxyl position [16]. The water solubilities of UA derivatives were significantly improved. UAMMC9 exhibited the best water solubility (39.7 μg/mL) (Figure 6). The studies with IGROV1 CR cells showed that UA and UAMMC9 act synergistically with cisplatin in inhibiting resistant cell growth. In vivo studies have also shown that treatments with cisplatin and UA or UAMMC9 exhibited a remarkable synergy in reducing tumor growth in nude mice compared to UA, UAMMC9, or cisplatin alone. Importantly, the treatments had minor toxicity. The UAMMC9 derivative inhibited tumor growth to a similar extent as UA, even when used at a dose 5-fold lower than UA. Experiments described by Zhang et al. indicate that UA and UAMMC9 bind directly to the catalytic site of SENP1, inhibit its activity at nM concentrations, and overcome ovarian cancer cisplatin resistance [16].

Twenty-nine commercially available natural ursane-type aglycones were tested for their SENP1 inhibitory activities, among which twelve aglycones showed IC_50_ activity at a concentration below 5 μM [17]. Pomolic acid and tormentic acid (Figure 5) were identified as potent SENP1 inhibitors with the IC_50_ values of 5.1 and 4.3 μM, respectively. Moreover, the combinations of cisplatin with pomolic acid (IC_50_ = 3.69 μM, combination index (CI) = 0.23) and tormentic acid (IC_50_ = 2.40 μM, CI = 0.30) exhibited potent platinum-resistant reversal activities compared to cisplatin only (IC_50_ = 28.23 μM) against the human ovarian cancer SKOV3 cells. The data suggested that pomolic acid and tormentic acid may be promising compounds for in vivo studies of platinum-resistant ovarian cancer with SENP1 activation [17].

A series of pentacyclic triterpenoid derivatives with hydrophilic or basic sidechain moieties introduced to the 28-carboxyl group of UA was designed and synthesized by Wei et al. [18]. Among these derivatives, ten pentacyclic triterpenoid derivatives exhibited superior activities to UA. Furthermore, the radiosensitization enhancement ratio (SER) was confirmed to be positively correlated. One of these derivatives, compound **2** in Figure 6, demonstrated the best radiosensitizing effect with an SER value of 1.45. Sumoylation and desumoylation play crucial roles in the DNA damage response and the development of resistance to radiotherapy. Inhibiting SENP1 upregulates the radiosensitivity of cancer cells, making it a promising target for radiosensitization. The studies presented by Wei et al. constitute the first report of small molecule SENP1 inhibitors with radiosensitizing activity [18].

Recently, Michalak et al. designed and synthesized twenty hybrids of UA with uracil, thymine, 6-methyluracil, and 2-thiouracil, in which UA was linked to the respective nucleobases via alkyl linkers of different carbon chain lengths (n = 4 or n = 6) at the C-28 position of the α-amyrin core [60]. The cytotoxic activity of the synthesized conjugates was determined using the hormone-dependent breast cancer cell line MCF-7, the triple-negative breast cancer (TNBC) cell line MDA-MB-231, and normal cell lines: human skin fibroblasts (CCD-25Sk) and human bronchial epithelium (BEAS-2B). The cytotoxicity studies of these hybrid compounds indicated that the five hybrids of UA with uracil derivatives exhibit a significant reduction in the cell viability of human breast cancer cell lines. One of them, compound **3** in Figure 6, demonstrated high cytotoxicity against MCF-7 and MDA-MB-231 cell lines with IC_50_ values of 14.00 µM and 5.83 µM, respectively. Biochemical studies have shown that this hybrid increased p53 and Bax protein levels in MDA-MB-231 cells, as well as significantly decreased Akt kinase levels, and efficiently inhibited collagen biosynthesis. These data indicate that the C-28 position, as well as the C-3 and C-17 positions, play an important role in the biological activity of UA and are associated with the increased anticancer activity of this compound and its derivatives [58,60,61].

### 3.5. Streptonigrin

**Figure 7:**
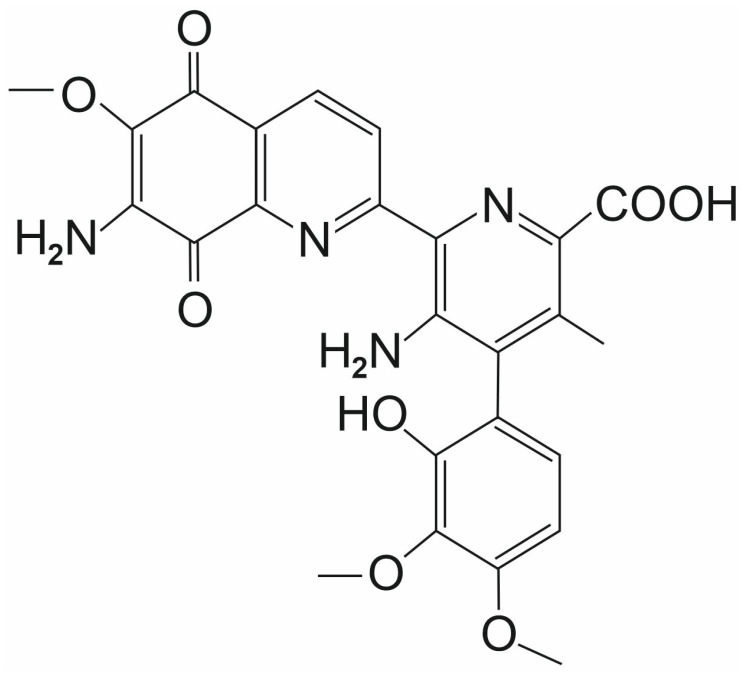
The chemical structure of streptonigrin (SN).

Streptonigrin (SN) is a naturally occurring an aminoquinone antibiotic isolated from the soil bacterium *Streptomyces flocculus*, known for its anticancer properties against a wide range of tumors [62,63,64,65] (Figure 7). Molecular docking and enzymatic activity analysis identified SN as one of the best possible SENP1/SUMO1 inhibitors among 260,000 small-molecule compound structures collected by the NCI Developmental Therapeutics Program [66].

In their experiment, Ambaye et al. [67] investigated the mechanism of SENP1 and SUMO1 interaction in the presence of SN. SN interacts directly with SENP1 through a binding site encompassing two key protein segments—the first consisting of seven amino acids at positions 494–500 and the second consisting of two amino acids at positions 511–512. The binding of SN disrupts the ability of SENP1 and SUMO1 to form a complex, which prevents the proper functioning of proteins and metabolic processes related to protein sumoylation and desumoylation. Asn494, Phe496, Lys500, Trp512, and Arg511 play a key role in the binding strength of SN to SENP1. The amino acid side chains interact directly with SN. Trp512 stabilizes this binding. The side chain of Trp512 interacts with the bicyclic quinoline ring, forming a pi stacking interaction. Additionally, Arg511 sidechain interacts with the methoxy group and forms a hydrogen bond with the carbonyl group of SN. Phe496 interacts with the phenyl ring and the methyl group on the pyridyl part of SN. Sidechain of Asn494 is responsible for binding to the NH_2_ group on the pyridyl ring by a hydrogen bond. Electrostatic interaction enabling the connection of the Lys500 sidechain with the carboxylic acid group in the SN molecule.

In an experiment involving the SENP1/SUMO1 complex, the unique inhibitory potential of SN was proven. In addition to inhibitory effect on SENP1, SN also inhibits the activity of other SENPs, but its inhibitory potential is significantly higher than that of other SENPs. For example, in the maturation reaction of SUMO1 and SUMO2 and their removal from protein conjugates, the SN IC_50_ for SENP1 was exceptionally low at 0.518 ± 0.100 μM compared to IC_50_ = 6.919 ± 0.676 μM for SENP2 [67].

The same group also investigated the inhibitory potential of SN on SENP1 in vitro using human colorectal carcinoma HCT-116 cells [67]. The effect of SN on the SENP1-dependent stability of HIF1α was investigated. HIF1α promotes angiogenesis by regulating dozens of proteins in cancer cells. SENP1 activity ensures the stability of the HIF1 molecule by preventing its sumoylation and proteasomal degradation. The presence of SN in the SENP1/SUMO1 complex disrupts its structure, leading to limited HIF1 desumoylation and, consequently, its degradation. This mechanism makes SN a promising compound in cancer therapies based on preventing the formation of new blood vessels that nourish rapidly dividing cancer cells [67].

### 3.6. Vialinin A and Thelephantin G

**Figure 8:**
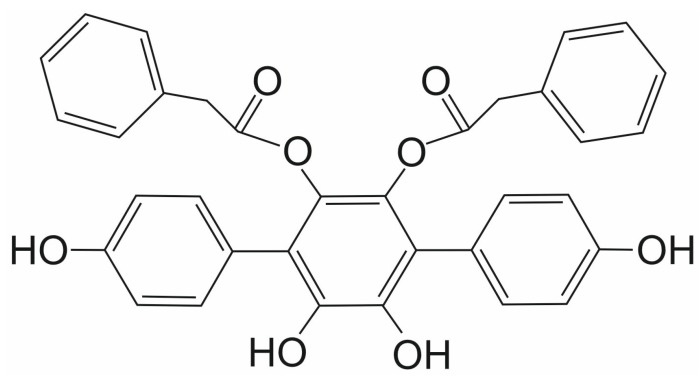
The chemical structure of vialinin A (VA).

**Figure 9:**
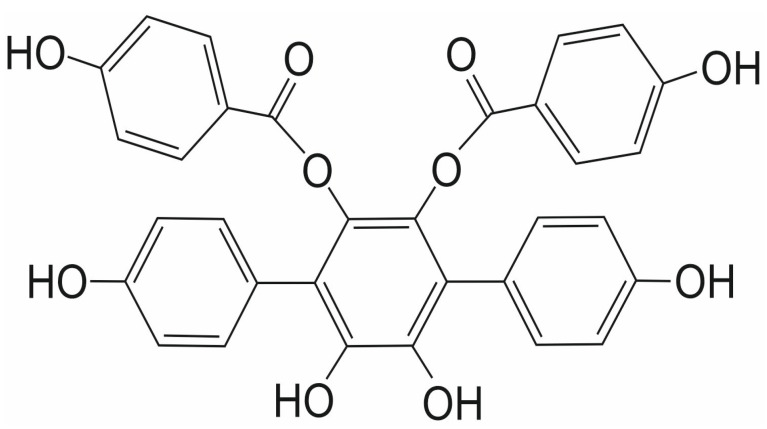
The chemical structure of thelephantin G (TG).

Vialinin A (VA) and thelephantin G (TG) are p-terphenyl compounds isolated from the mushroom *Thelephora vialis* (Figure 8 and Figure 9, respectively). They are both known primarily as potent inhibitors of ubiquitin-specific peptidase 5, which is involved in various cellular processes, including DNA repair, inflammation, and stress response, as well as cancer initiation and progression [68,69,70,71,72,73,74,75]. Both compounds belong to a promising group of p-terphenyl drugs isolated from fungi of the Thelephora genus.

Their activity as SENP1 inhibitors has been investigated in only one study to date. Both compounds differ slightly in their inhibitory effect against the isopeptidase activity of SENP1 [76]. The Yoshioka et al. group examined the enzymatic activity of both compounds against the catalytic domain of the human SENP1 (cSENP1) protein. The inhibitory IC_50_ values obtained for cSENP1 were 1.89 ± 0.04 μM for VA and 1.52 ± 0.06 μM for TG. In an identical experiment concerning recombinant full-length human SENP1 (rfSENP1) obtained from SENP1 total RNA derived from human basophilic leukemia KU-812 cells, the estimated inhibitory IC_50_ value for VA was 1.64 ± 0.23 μM. In contrast, for TG, it was higher, at 2.48 ± 0.02 μM [76].

Based on inhibitory experiments performed with cellular extracts, Yoshioka et al. selected SENP1 as the primary target for VA and TG in human KU-812 cells. They also postulated that a phenylacetyl group in VA (Figure 8), a benzoic group in TG (Figure 9), and two hydroxyl groups at the catechol structure of the benzene ring are crucial for the inhibitory activity of both compounds against SENP1 [76].

### 3.7. Other Natural SENP1 Inhibitors

**Figure 10:**
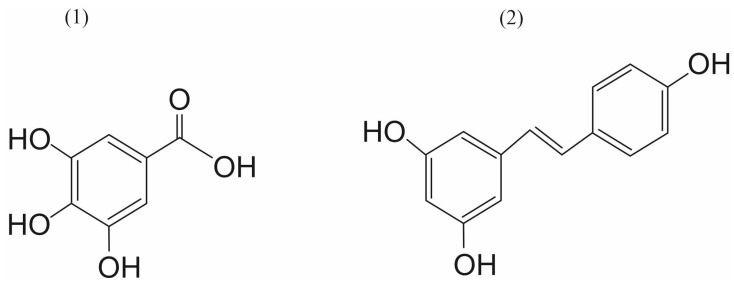
The chemical structures of gallic acid (**1**) and resveratrol (**2**).

Molecular docking of SENP1 with natural compounds, including gallic acid, caffeic acid, thymoquinone, thymol, betaine, alkannin, ellagic acid, betanin, shikonin, betanidin, and momordin IC was performed using AutoDock 4. Following docking, complexes were subjected to molecular dynamics (MD) simulation with GROMACS 4.6.5. Among the tested compounds, gallic acid yielded the most significant results, with high stability, elevated hydrogen bonding, high binding energy, and the strongest intermolecular interactions with SENP1 (Figure 10). Compared to Mc, which served as a control, gallic acid demonstrated stronger interactions and a more favorable toxicity profile. Notably, gallic acid is a phenolic compound known to affect several pharmacological and biochemical pathways, exhibiting potent antioxidant, anti-inflammatory, antimutagenic, and anticancer properties [77]. Building on these findings, further research could significantly improve the application of plant products. Basic, preclinical, and clinical research on gallic acid may provide a roadmap for its potential use in cancer prevention [78].

Taghvaei et al. also screened the ZINC database library and evaluated a wide range of natural compounds for their ability to inhibit SENP1. In this research, computational tools—including ADME/Tox property analysis, ligand-based virtual screening, and molecular dynamics were essential. The results indicate that resveratrol (among the selected compounds) (Figure 10) and ZINC33916875 (from the ZINC database) could be the most promising SENP1 inhibitory ligands [79].

## 4. Conclusions and Prospects for Further Research

The naturally derived SENP1 inhibitors presented in this paper—momordin Ic (Mc), hinokiflavone (HNK), triptolide (TPL), ursolic acid (UA), streptonigrin (SN), vialinin A (VA), and thelephantin G (TG), exhibit broad anticancer activity by affecting numerous proteins, signaling pathways, and cellular processes. Numerous studies have also demonstrated their synergistic effects with classic anticancer drugs such as cisplatin. Furthermore, and this is particularly important from the perspective of anticancer therapy, these compounds can sensitize resistant cancer cells to drugs and radiotherapy [17,18,49,50,51]. Natural SENP1 inhibitors currently constitute a small group of well-studied compounds. However, given the properties of the compounds described in this paper, it is worth emphasizing that the search for new compounds with anticancer activity associated with SENP1 inhibition is ongoing. This search is multifaceted. On the one hand, the plant world is being scanned for compounds with appropriate structures and properties, which are then tested in extracellular and cellular models, as well as in vivo. Another approach involves chemical modifications of known compounds, resulting in new molecules with improved properties, such as better water solubility or reduced systemic toxicity. To increase the bioavailability of natural SENP1 inhibitors, they are combined with various carriers, such as TPL solid lipid nanoparticles (TCM), pH-stimulated responsive nanocarriers, or transferrin-modified liposomes with TPL (Tf-Tp@Lip) [80]. Yet another approach involves combining two natural compounds, e.g., Mc and gallic acid. The combination of Mc and gallic acid, both enriched in the extract of *Momordica charantia*, significantly eliminates both normoxic active and hypoxic dormant tumor cells. It is a promising lead in anticancer drug development [32]. *Momordica charantia* is in Phase II clinical trials. It is already a certified nutraceutical-based Ayurvedic medicine, a form of medical practice prevalent in India and Asia [32].

Research results presented in this review indicate that inhibiting SENP1 activity may be an effective new targeted anticancer therapy. Naturally derived SENP1 inhibitors have great potential in this regard.

## Data Availability

No new data were created or analyzed in this study. Data sharing is not applicable to this article.

## Abbreviations

ADAM10	metalloprotease-10
AKT	protein kinase B (PKB)
AML	acute myeloid leukemia
AR	androgen receptor
CDK	cyclin-dependent kinase
CESTA	cellular thermal shift assay
CI	combination index
COX-2	cyclooxygenase 2
DARTS	drug affinity responsive target stability
DCTP1	dCTP pyro-phosphatase 1
DNA-PKcs	DNA-dependent protein kinase catalytic subunit
HDAC1	histone deacetylase 1
HIF1α	hypoxia-inducible factor 1-alpha
HNK	Hinokiflavone
HO-1	heme oxygenase 1
HSP70	70 kDa heat shock protein
IC_50_	half maximal inhibitory concentration
iNOS	inducible nitric oxide synthase
JAK2	Janus kinase 2
MAPK	mitogen-activated protein kinase
Mc	momordin Ic
MCL1	Myeloid Cell Leukemia 1 protein
MCL1-L	long isoform of Myeloid Cell Leukemia 1 protein
MCL1-S	short isoform of Myeloid Cell Leukemia 1 protein
MDM2	mouse double minute 2 homolog
MMPs	matrix metalloproteinases
MUTYH	DNA glycosylase
NF-κB	nuclear factor kappa-light-chain-enhancer of activated B cells
OGG1	8-oxoguanine glycosylase
p300	histone acetyltransferase
PC-2	polycystin-2
PIAS proteins	protein inhibitors of activated signal transducer and activator of transcription
PI3K	phosphoinositide 3-kinase
PPARγ	peroxisome proliferator-activated receptor gamma
Prx-I	peroxiredoxin-I
rfSENP1	recombinant full-length human SENP1
ROS	reactive oxygen species
RUNX2	Runt-related transcription factor 2
SAE1 and SAE2	SUMO-activating enzymes 1 and 2
SENP	sentrin-specific protease
SER	radiosensitization enhancement ratio
SN	streptonigrin
SRC-1	steroid receptor coactivator-1
SRC-2/GRIP1	steroid receptor coactivator-2/glucocorticoid receptor-interacting protein 1
SUMO	small ubiquitin-like modifier
TAB1	TAK1 binding protein
TAK1	transforming growth factor-β-activated kinase 1
TCM	TPL solid lipid nanoparticles
Tf-Tp@Lip	transferrin-modified liposomes with TPL
TG	thelephantin G
TPL	triptolide
UA	ursolic acid
UBC9	SUMO conjugating enzyme E2
VA	vialinin A
XPB or ERCC3	xeroderma-pigmentosum B or ERCC excision repair 3 protein
XRCC1	X-ray repair cross-complementing protein 1, DNA repair protein 1
